# The Kinase Activity-deficient Isoform of the Protein Araf Antagonizes Ras/Mitogen-activated Protein Kinase (Ras/MAPK) Signaling in the Zebrafish Embryo*

**DOI:** 10.1074/jbc.M115.676726

**Published:** 2015-08-25

**Authors:** Cong Xiong, Xingfeng Liu, Anming Meng

**Affiliations:** From the State-Key Laboratory of Membrane Biology, Tsinghua-Peking Center for Life Sciences, School of Life Sciences, Tsinghua University, Beijing 10084, China

**Keywords:** embryo, fibroblast growth factor (FGF), mitogen-activated protein kinase (MAPK), Ras protein, zebrafish, Araf, mesoderm, patterning, transcript variant

## Abstract

Raf kinases are important components of the Ras-Raf-Mek-Erk pathway and also cross-talk with other signaling pathways. Araf kinase has been demonstrated to inhibit TGF-β/Smad2 signaling by directly phosphorylating and accelerating degradation of activated Smad2. In this study, we show that the *araf* gene expresses in zebrafish embryos to produce a shorter transcript variant, *araf-tv2*, in addition to the full-length variant *araf-tv1. araf-tv2* is predicted to encode a C-terminally truncated peptide without the kinase activity domain. Araf-tv2 can physically associate with Araf-tv1 but does not antagonize the inhibitory effect of Araf-tv1 on TGF-β/Smad2 signaling. Instead, Araf-tv2 interacts strongly with Kras and Nras, ultimately blocking MAPK activation by these Ras proteins. In zebrafish embryos, overexpression of *araf-tv2* is sufficient to inhibit Fgf/Ras-promoted Erk activation, mesodermal induction, dorsal development, and neuroectodermal posteriorization. Therefore, different isoforms of Araf may participate in similar developmental processes but by regulating different signaling pathways.

## Teaser

**Background:** The full-length Araf (Araf-tv1) inhibits Nodal/Smad2-induced mesendodermal induction in zebrafish embryos.

**Results:** Araf-tv2, a kinase-deficient isoform of zebrafish Araf, can inhibit developmental functions of Fgf/Ras signaling.

**Conclusion:** Araf-tv1 and Araf-tv2 regulate distinct signaling pathways in zebrafish embryos.

**Significance:** The zebrafish *araf* gene is expressed to produce different variants with distinct functions during embryogenesis.

## Introduction

Germ layer specification and patterning are important events of early embryonic development and are directed by multiple signaling pathways. Nodal signal is the key mesendoderm inducer and dorsalizing signal during amphibian and fish embryogenesis (1–5). Studies in the zebrafish indicate that maternal Wnt/β-catenin signaling is essential for initiating the expression of zygotic nodal genes in the dorsal blastodermal margin (2) while maternal Eomesodermin is required for nodal genes expression in the lateral and ventral blastodermal margin (6). Fibroblast growth factors (FGFs)^2^ in part mediate Nodal activity in mesendoderm induction and dorsal development and the zygotic expression of *fgf* genes requires Nodal and Wnt/β-catenin signals (7–11).

The Ras-Raf-Mek-Erk kinase cascade is one of the most important pathways downstream of Fgf signaling (12). Each component of the cascade has multiple family members, *e.g.* Nras, Kras, and Hras of the Ras family, Araf, Braf, and Raf1 of the Raf family, Mek1 and Mek2 of the Mek family, and Erk1 and Erk2 of the Erk family. During signaling transduction, the membrane-localized GTP-bound Ras, which is activated by cytokine growth hormones, targets Raf for phosphorylation; the activated Raf kinase activates MEK1/2 via phosphorylation; and the activated Mek1/2 in turn phosphorylate Erk1/2. This cascade has been shown to participate in regulation of various cellular processes, such as cell differentiation, proliferation, migration and survival, and diseases (13, 14).

Recently, we demonstrate that Araf directly cross-talks with Nodal/Smad2 signaling in a MAPK-independent fashion (15). Full-length Araf inhibits Nodal/Smad2 signaling by directly phosphorylating the linker region of Smad2, leading to ubiquitination and degradation of activated Smad2. In the zebrafish embryo, *araf* antagonizes mesendoderm induction and dorsalization exerted by Nodal signal. Yokoyama *et al.* reported that the murine *Araf* locus could express a truncated isoform, DA-Raf1, which retains the Ras-binding domain but lacks the kinase domain (16). DA-Raf1 was found to intervene in ERK activation and positively regulate myogenic differentiation. It is unclear whether the zebrafish *araf* locus could produce similar truncated isoforms with a role in embryonic development.

In this study, we identified the *araf* transcript variant *araf-tv2* in the zebrafish embryo, which is predicted to encode a C-terminally truncated protein with loss of the kinase activity domain. Although Araf-tv2 is able to physically associate with full-length Araf (Araf-tv1), it does not interfere with inhibitory effect of Araf-tv1 on Nodal/Smad2 signaling. Instead, Araf-tv2 inhibits Ras/MAPK pathway and may play a role in embryonic development by controlling Fgf/MAPK signaling.

## Experimental Procedures

### 

#### 

##### Zebrafish Strains

Wild-type Tübingen strain was used in this study. Embryos were raised in Holtfreter's water at standard 28.5 °C as previously described (17). Ethical approval was obtained from the Animal Care and Use Committee of Tsinghua University.

##### Plasmids

Zebrafish *araf-tv2, kras, hras*, and *nras* were amplified from the cDNA pool of zebrafish embryos. The coding sequence of human *HRAS*, *KRAS*, or *NRAS* was amplified from cDNAs of HEK293T cells. For synthesizing mRNA, the coding sequence of the corresponding gene was cloned into *pXT7* or *pCS2* vector; for transfection in mammalian cells, the coding sequence was cloned into *pCMV5* vector with HA, Myc, or Flag tag. Human or zebrafish Hras G12V, Kras G12V, or Nras G12V constructs were made by mutating the 12^th^ glycine to valine. HA-tagged Araf, Araf-N, Araf-C constructs were described before (15). To make *araf-tv2* specific antisense RNA probe, a fragment contained 314 bp of *araf-tv2* 3′UTR was amplified and subcloned into *EZ-T* vector.

##### mRNA Synthesis and Microinjection

mRNAs were synthesized *in vitro* using T7 or SP6 mMessage mMachine Kit (Ambion) and purified using the Qiagen's RNeasy Mini Kit. Individual or mixed mRNAs were injected into the yolk of zebrafish embryos at the one-cell stage. araf-MOs were described previously (15).

##### Reverse Transcription and PCR

Total RNAs were isolated from embryos at desired stages and used for reverse transcription using oligo(T)_18_ or specific primers. The sequence consisting of 3′ coding and 3′ untranslated regions of *araf-tv2* was amplified by PCR using p1/pT_15_VV primers. Specific regions of *araf-tv1* and *araf-tv2* were amplified by PCR using p1/p3 primers for *araf-tv1* and p4/p5 primers for *araf-tv2* in the same reaction tube. β-actin was amplified as the internal control. Primer sequences were: p1, 5′-CGAATATCAACAACCGCGATCAGAT-3′; p2, 5′-TTCACATGGTCAGCACGCTGGA-3′; p3, 5′-TAACTAGGGTGCTCTCAGGAATGTG-3′; p4, 5′-CTAAAAGCTCAAATCCACATCTCTT-3′; p5, 5′-CTTTTCCAAACAATATATGCTAATA-3′; pT_15_VV, 5′-TAGCAGGTCCAG(T)_15_VV-3′ (V = G, A, or C); upper primer for β-actin, 5′-ATGGATGATGAAATTGCCGCAC-3′; and lower primer for β-actin, 5′-ACCATCACCAGAGTCCATCACG-3′. The amplified products were resolved on agarose gels by electrophoresis. The relative band intensity was analyzed by Image J software.

##### Whole-mount in Situ Hybridization and Immunofluorescence

Antisense RNA probes were made by *in vitro* transcription using digoxigenin-labeled UTP. Whole-mount *in situ* hybridization was performed following standard procedures. Endogenous p-Erk1/2 was detected by whole-mount immunofluorescence using p-Erk1/2 antibody (CST #9101, diluted in 1: 200) as described before (15). The embryos were observed by confocal microscopy.

##### Cell Culture, Transfection, Luciferase Reporter Assay, Immunoblotting, and Co-immunoprecipitation

HEK293T or Hep3Bcells were cultured in DMEM or MEM (Gibco) medium containing 10% fetal bovine serum (Hyclone). All assays were performed as described before (15). The used primary antibodies were: anti-Myc and anti-HA from Santa Cruz; anti-Flag from Sigma; anti-Smad2/3, anti-phospho-Smad2(Ser-465/467), and anti-phospho-ERK1/2 (Thr-202/Tyr-204) from Cell Signaling Technology; anti-ERK1 from OriGene; anti-pAKT (Ser-473) and anti-p-Jun1/2(Thr-183/Tyr-185) from Cell Signaling Technology, anti-β-actin from Santa Cruz, and anti-α-tubulin from EasyBio.

## Results

### 

#### 

##### araf-tv2 Is Expressed during Zebrafish Early Embryonic Development

**FIGURE 1.**
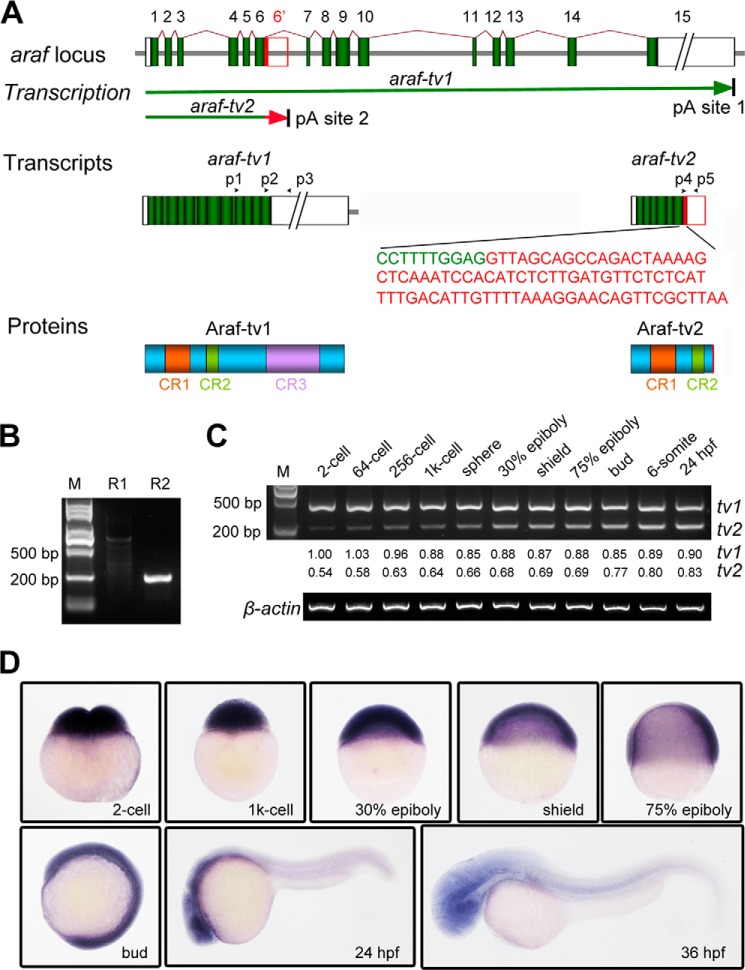
**Generation and expression pattern of *araf-tv2* variant.** *A*, schematic diagrams of *araf* expression products. The zebrafish *araf* locus consists of 15 exons (1–15). The transcription would halt at the alternative polyadenylation (pA) sites, ultimately generating *araf-tv1* and *araf-tv2*. The 84-bp coding sequence specific for *araf-tv2* was shown in red letters. The *boxes* in transcripts represented untranslated regions. The *arrowheads* indicated primers used. *B*, amplification of *araf-tv2* transcript. For the 1st round (R1) and 2nd round (R2) RT-PCR, P1/pT_15_VV, and p2/p5 primer pairs were used, respectively. The product in R2 was confirmed by sequencing. *M*, molecular markers. *C*, relative amounts of *araf-tv1* and *araf-tv2* transcripts in embryos at different stages. The p1/p3 primers for *araf-tv1* and p4/p5 primers for *araf-tv2* were mixed for amplifying both transcripts in the same reaction tube by semi-quantitative RT-PCR. β-actin was used as the internal control. The bands resolved in the gel were quantified by Image J software. *D,* expression pattern of *araf-tv2* in zebrafish embryos were detected by whole mount *in situ* hybridization.

The mouse *Araf* gene has been found to express a C-terminally truncated Araf isoform, which lacks the kinase activity domain, in addition to the full-length Araf (16) (18). According to ZFIN, the zebrafish *araf* locus consists of 15 exons and 14 introns and is predicted to produce three *araf* transcript variants (Fig. 1*A*). The two long variants encode an identical full-length Araf protein of 608 residues and were then named *araf-tv1* thereafter; the third variant is shorter, expected to code for a C-terminally truncated Araf protein of 265 residues, and named *araf-tv2* (Fig. 1*A*). Compared with *araf-tv1*, *araf-tv2* contains a sequence derived from the 6th intron, which is immediately downstream of the sequence of the 6th exon, and does not harbor any sequences from 7th-15th exons. The variants *araf-tv1* and *araf-tv2* are most likely to be generated by alternative cleavage and polyadenylation because they have a completely different 3′ untranslated region. The zebrafish Araf-tv2 protein is similar to the murine DA-Raf1 (16), which lacks the CR3 domain of kinase activity.

The existence of *araf-tv2* in zebrafish embryos was confirmed by two rounds of RT-PCR using total RNA isolating from embryos at 24 hpf as template (Fig. 1*B*), which was followed by sequencing. Using *araf-tv1* and *araf-tv2* specific primers, we simultaneously detected both transcript variants in embryos at different stages by RT-PCR in the same reaction mixture. Results showed that the *araf-tv1* level remained high from the 2-cell stage to 24 h postfertilization (hpf), whereas the *araf-tv2* level was low at the 2-cell stage and then gradually increased (Fig. 1*C*). However, the *araf-tv2* levels were always lower than the *araf-tv1* levels before the completion of gastrulation (bud stage). Whole-mount *in situ* hybridization using *araf-tv2* specific probe revealed that *araf-tv2* transcripts were ubiquitously distributed in embryos from 2-cell to bud stages and mainly enriched in the head region at 24 and 36 hpf (Fig. 1*D*), which resembled *araf-tv1* expression patterns (15). The expression pattern of *araf-tv2* suggests a role in early development of zebrafish embryos.

##### Araf-tv2 Hardly Intervenes with Araf-tv1 Functions in Vitro

**FIGURE 2.**
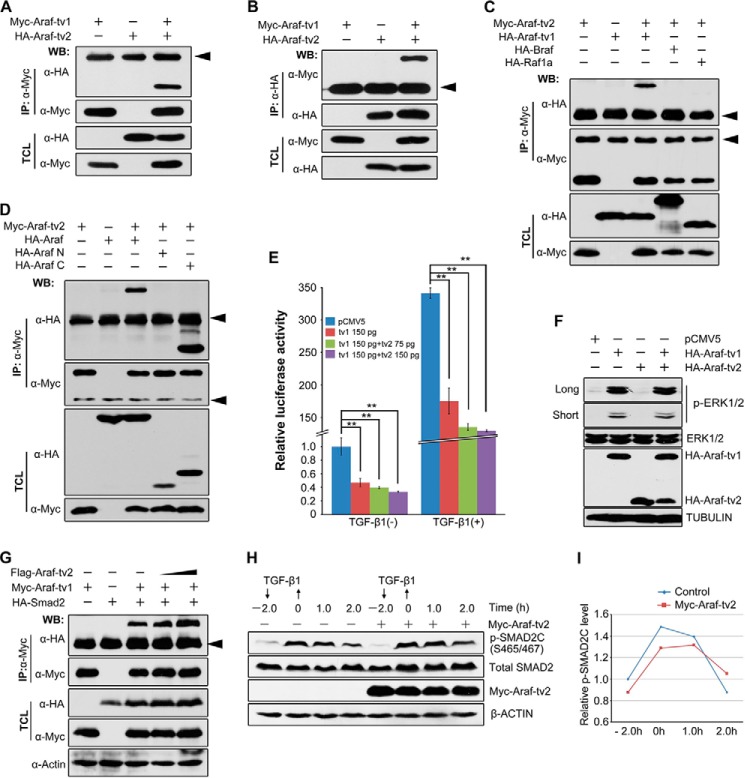
**Araf-tv2 interacts with but does not antagonize functions of Araf-tv1.** *A* and *B*, detection of Araf-tv1 and Araf-tv2 interactions by co-immunoprecipitation (co-IP). Tagged Araf-tv1 and Araf-tv2 were co-expressed in HEK293T cells. *IP*, immunoprecipitation; *WB*, Western blot; *TCL*, total cell lysates. *Arrowheads* indicated the nonspecific IgG band (same for other panels). *C,* detection of physical interaction between Araf-tv2 and different Raf members by Co-IP in HEK293T cells. Note that Araf-tv1 only associated with Araf-tv1 but not with Braf or Raf1a. *D,* Araf-tv2 interacted with the C-terminal part of Araf-tv1. Araf N and Araf C referred to N-terminal and C-terminal (containing the kinase domain) parts of Araf, respectively. *E,* Araf-tv2 did not antagonize the inhibitory effects of Araf-tv1 on ARE_3_-luciferase reporter expression with or without TGF-β1 stimulation in Hep3B cells. The indicated amount of plasmids was for each well in a 24-well plate. Statistical significance: **, *p* < 0.01 by Student's *t* test. *F,* Araf-tv2 could not block Araf-tv1-stimulated ERK activation in HEK293T cells. Long and short referred to longer and shorter exposure time, respectively. Endogenous ERK1/2, p-ERK1/2, and tubulin were examined. *G,* Araf-tv2 didn't interfere with Araf-tv1 interaction with Smad2. *H* and *I,* Araf-tv2 didn't promote the degradation of p-SMAD2C in Hep3B cells. Endogenous p-SMAD2C was detected using anti-p-SMAD2 (S465/467) antibody (*H*). The relative p-SMAD2C level was the ratio of p-SMAD2C to total SMAD2, which was measured by Image J analysis (*I*).

We previously demonstrated that zebrafish full-length Araf, *i.e.* Araf-tv1, acts to inhibit TGF-β/Smad2 signaling and Nodal-dependent mesendodermal induction (15). We asked whether Araf-tv2 could antagonize Araf-tv1 function as a dominant negative form. As the first step, we investigated their physical interaction. Immunoprecipitation results showed that Araf-tv2 bound to Araf-tv1 (Fig. 2, *A* and *B*) but not to Braf or Raf1a (Fig. 2*C*) in HEK293T cells, and that Araf-tv2 associated with the kinase domain-containing C-terminal part of Araf-tv1 (Fig. 2*D*).

Then, we tested whether Araf-tv2 could exert an effect on the inhibitory role of Araf-tv1 in TGF-β/Smad2 signaling. When Araf-tv1 was transfected into Hep3B cells, the expression of the Smad2-responsive reporter ARE-luc was inhibited no matter whether exogenous TGF-β1 was added or not (Fig. 2*E*). The co-expression of Araf-tv2 was unable to release Araf-tv1 inhibition of the reporter expression (Fig. 2*E*). Further biochemical analyses demonstrated that that Araf-tv2 neither interfered in physical interaction between Araf-tv1 and Smad2 in HEK293T cells (Fig. 2*G*) nor attenuated Araf-tv1-promoted degradation of activated SMAD2 (p-SMAD2C) in Hep3B cells (Fig. 2, *H* and *I*). These data indicate that Araf-tv2 does not antagonize Araf-tv1 function in inhibiting TGF-β/Smad2 signaling.

In the Ras-Raf-Mek-Erk cascade, Araf could stimulate downstream MAPK activation (19). In HEK293T cells, the basal level of p-ERK1/2 was low; the p-ERK1/2 level was up-regulated drastically by Araf-tv1 transfection but decreased slightly by Araf-tv2 transfection (Fig. 2*F*). Interestingly, Araf-tv1-induced p-ERK1/2 activation in HEK293T cells was not blocked by co-transfection of Araf-tv2. This result implies that Araf-tv2 may not intervene in Araf-mediated MAPK activation, at least in cultured cells.

##### Araf-tv2 Inhibits FGF/MAPK Signaling in Zebrafish Embryos

**FIGURE 3.**
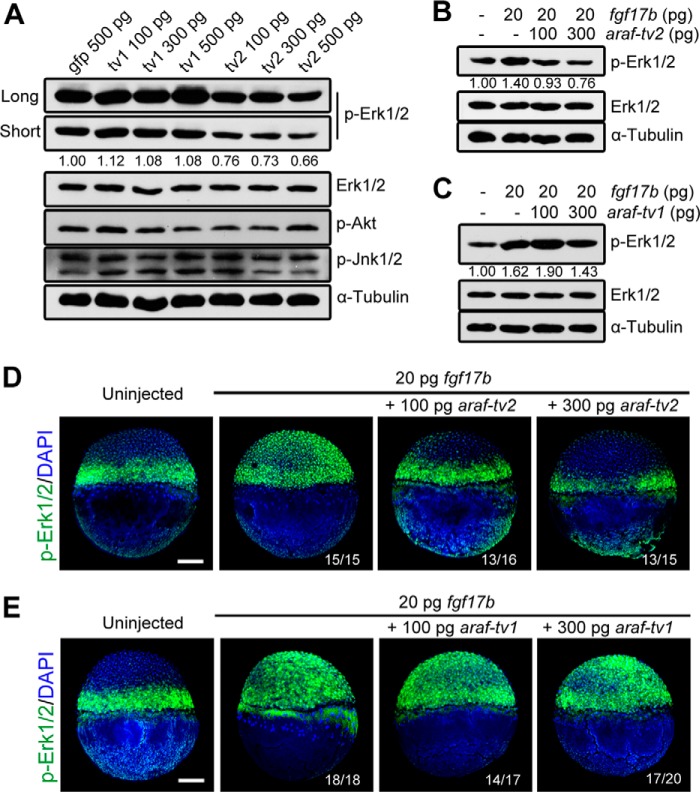
**Araf-tv2 attenuates Fgf-stimulated MAPK activation in zebrafish embryos.** *A*, overexpression of *araf-tv2* mRNA decreased p-Erk1/2 levels in embryos. Embryos at the one-cell stage were injected with three increasing doses of *araf-tv1* or *araf-tv2* mRNA and collected at 75% epiboly stage for immunoblotting using different primary antibodies. The control embryos were injected with *gfp* mRNA. The bands of p-Erk1/2 were quantified by Image J software. *B* and *C*, p-Erk1/2 was detected by immunoblotting in embryos injected with *fgf17b* mRNA alone or in combination with *araf-tv2* (*B*) or *araf-tv1* mRNA (*C*) The p-Erk1/2 levels were analyzed. *D* and *E*, spatial distribution of p-Erk1/2 was detected by immunofluorescence in embryos injected differently. Ratios of affected embryos were indicated. *Scale bar*, 200 μm.

DA-Raf1, a splicing isoform of murine Araf without the kinase activity domain, is shown to antagonize Ras-Erk signaling (16). We wondered whether *araf-tv2* overexpression would impair MAPK activation in zebrafish embryos. As shown in Fig. 3*A*, the level of endogenous p-Erk1/2 was high in control embryos. However, *araf-tv2* overexpression caused an obvious reduction of p-ERK1/2 amount, which was in contrast to a marginal increase of p-Erk1/2 in embryos overexpressing *araf-tv1*. On the other hand, either of isoforms did not cause apparent change in the amount of p-Akt and p-Jnk1/2, which could be activated by cytokine growth hormones. Given that *fgf17b* is a potent regulator of zebrafish embryonic development (9), we tested whether *araf-tv2* overexpression had an impact on MAPK activation upon *fgf17b* overexpression. Western blot analysis using embryonic cell lysates disclosed that co-injection of *araf-tv2* and *fgf17b* mRNAs led to a marked decrease of p-Erk1/2 amount compared with *fgf17b* mRNA injection alone (Fig. 3*B*), whereas this effect was not observed for co-injection of *araf-tv1* and *fgf17b* mRNAs (Fig. 3*C*). As detected by immunofluorescence, p-Erk1/2 was essentially restricted to the blastodermal margin at the shield stage. Overexpression of *fgf17b* induced p-Erk1/2 throughout the entire blastoderm, and the ectopic p-Erk1/2 was wholly wiped away by co-overexpression of *araf-tv2* but not *araf-tv1* (Fig. 3, *D* and *E*). Taken together, these results suggest that *araf-tv1* and *araf-tv2* have different functions during development with *araf-tv2* antagonizing Fgf/MAPK signaling.

##### Both Araf-tv2 and Araf-tv1 Inhibit Germ Layer Formation and Patterning

**FIGURE 4.**
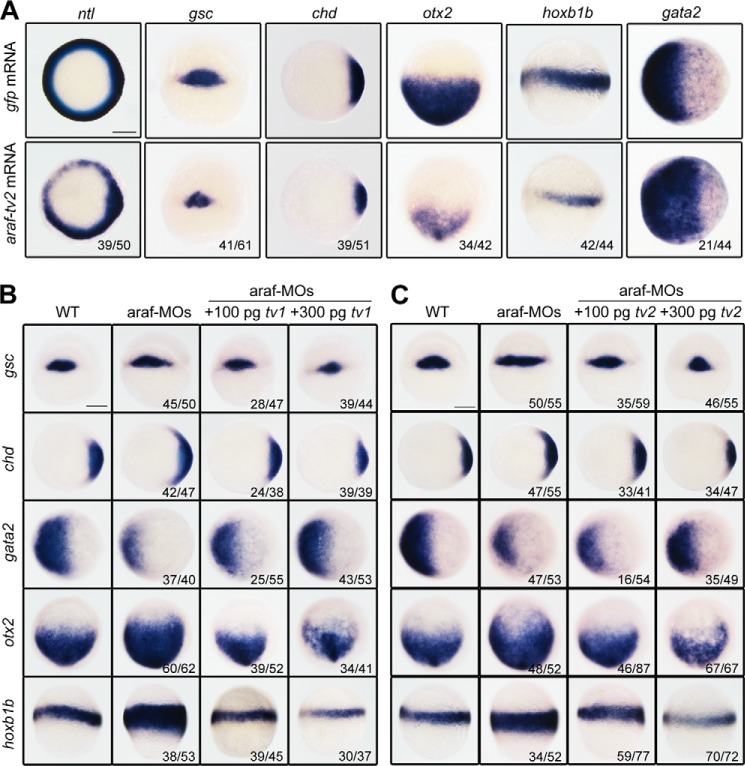
**Effect of *araf-tv2* overexpression on germ layer marker expression.** *A*, different germ layer markers were examined in embryos injected with 500 pg *araf-tv2* or *gfp* (control) mRNA. *B* and *C*, rescue effect of *araf-tv1* or *araf-tv2* on marker expression in *araf* morphants. Embryos at the one-cell stage were injected with 20 ng of araf-MO1/araf-MO2 (*araf-MOs*) alone or together with *araf-tv1* or *araf-tv2* mRNA and collected at later stages for *in situ* hybridization. Except that *otx2* and *hoxb1b* were examined at the 75% epiboly stage, the other markers were examined at the shield stage. Orientations of embryos: animal-pole views with dorsal to the right for *ntl*, *chd*, and *gata2*; animal-pole views with dorsal to the bottom for *otx2;* dorsal views for *gsc*; lateral views with dorsal to the right for *hoxb1b*. The ratio of embryos with the representative pattern was indicated. *Scale bar*, 200 μm.

Because of technical difficulty of specifically knocking down or knocking out *araf-tv2*, we investigated its possible function first by injecting *araf-tv2* mRNA into one-cell stage embryos and examining markers expression during gastrulation. Overexpression of *araf-tv2* resulted in a decrease of the expression of the pan-mesodermal marker *ntl* and the dorsal markers *gsc* and *chd* (Fig. 4*A*). These results suggest that, like *araf-tv1* (15), *araf-tv2* plays an inhibitory role in mesodermal induction and dorsal development. Furthermore, the *araf-tv2* injected embryos exhibited decreased expression of the anterior neuroectodermal marker *otx2* and the posterior neuroectodermal marker *hoxb1b* but had an expanded domain of the ventral epidermal marker *gata2* (Fig. 4*A*). It is likely that *araf-tv2* acts to repress neural induction. Interestingly, effect of *araf* knockdown could be efficiently compromised by either *araf-tv1* or *araf-tv2* overexpression (Fig. 4, *B* and *C*). The underlying mechanisms may be different because the two *araf* variants act on different signaling pathways.

##### Araf-tv2 Inhibits Fgf-induced Germ Layer Formation and Patterning

**FIGURE 5.**
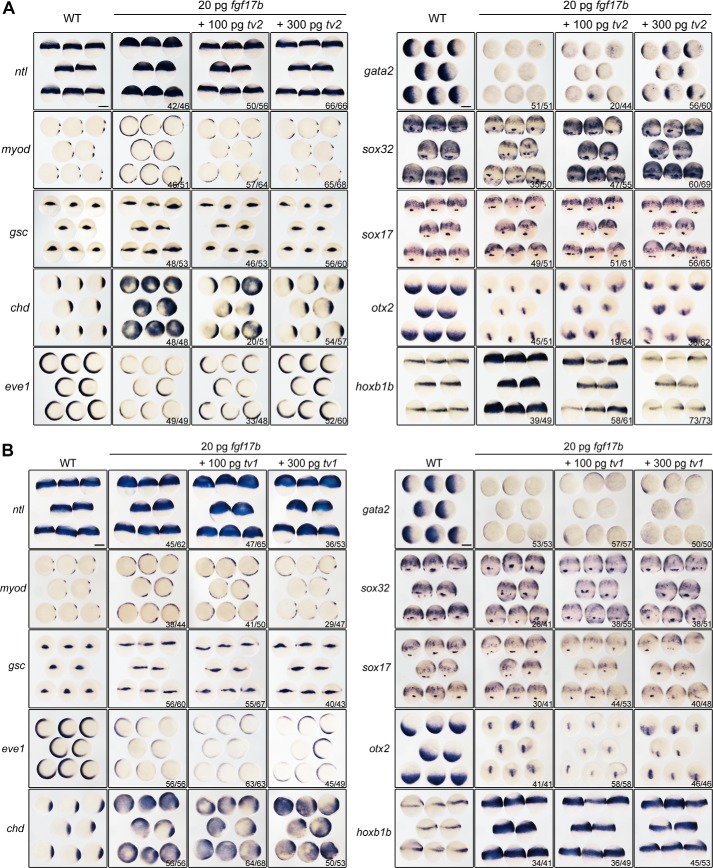
**araf-tv2 overexpression antagonizes *fgf17b*-induced marker changes.** *A* and *B,* expression patterns of marker genes revealed by whole-mount *in situ* hybridization. Embryos at the one-cell stage were injected with *fgf17b* mRNA alone or together with *araf-tv2* (*A*) or *araf-tv1* (*B*) mRNA and harvested for probing at the shield stage for *ntl*, *gsc*, *chd*, *eve1*, and *gata2* or at the 75% epiboly stage for *myod*, *sox32*, *sox17*, *otx2*, and *hoxb1b.* Orientations of embryos: lateral views for *ntl* and *hoxb1b*; animal-pole views with dorsal to the right for *myod*, *chd*, *eve1*, and *gata2*; dorsal views for *gsc*, *sox32*, and *sox17*; animal-pole views with dorsal to the bottom for *otx2*. The ratio of embryos with marker change was indicated. *Scale bar*, 200 μm.

Next, we tested whether *araf-tv2* could inhibit functions of Fgf signaling during embryonic development. As shown in Fig. 5*A*, injection of *fgf17b* mRNA alone in zebrafish embryos enhanced mesoderm induction and dorsal development with ectopic or expanded expression of *ntl, myod*, *gsc*, and *chd* and decreased expression of *eve1* and *gata2*, inhibited endoderm formation with a reduction of *sox32* and *sox17* expression, and promoted neuroectodermal posteriorization with an expanded *hoxb1b* expression domain and a smaller *otx2* expression domain. These effects were compromised by co-overexpression of *araf-tv2* in a dose-dependent manner. In sharp contrast, co-overexpression of *araf-tv1* had little effect on *fgf17b*-induced marker changes. Therefore, *araf-tv2* may regulate embryonic development mainly by inhibiting Fgf signaling, a mechanism differing from *araf-tv1* (15).

##### Araf-tv2 Inhibits Nras and Kras Signaling

**FIGURE 6.**
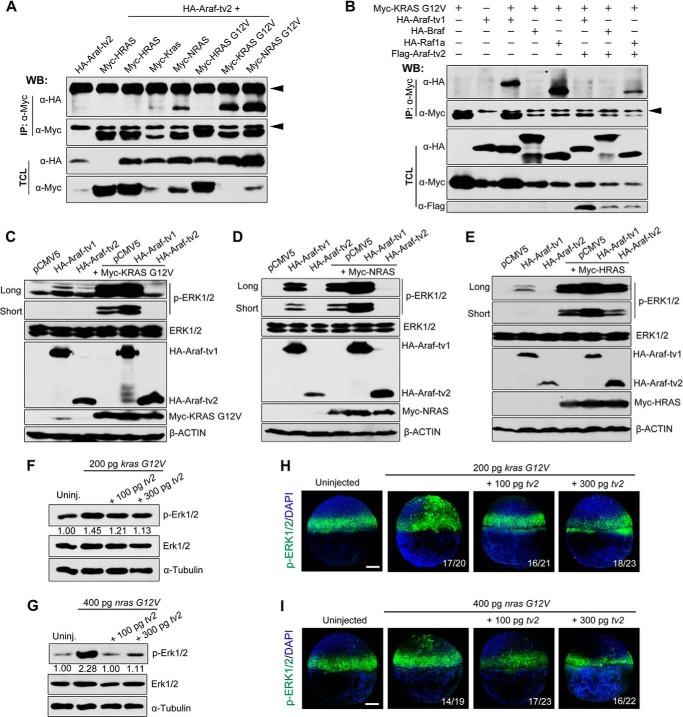
**Araf-tv2 binds to Ras proteins and inhibits Ras-stimulated MAPK activation.** *A*, Araf-tv2 bound to KRAS and NRAS. Interactions between Araf-tv2 and different human RAS proteins were detected in HEK293T cells by co-immunoprecipitation. The G12V mutation made the corresponding Ras constitutively active. *Arrowheads* indicated the nonspecific IgG. *B*, Araf-tv2 impaired the binding of Ras to Raf members in HEK29T cells. Note that KRAS G12V only interacted weakly with Braf as indicated by *. *C–E*, overexpression of Araf-tv2 attenuated RAS-induced increase of endogenous p-ERK1/2 in HEK293T cells. Note that Araf-tv2 efficiently blocked effect of KRAS G12V (*C*) and NRAS (*D*) but had a little effect on HRAS activation of p-ERK1/2 (*E*). Co-transfection of Araf-tv1 enhanced Ras activation of ERK1/2. *F* and *G, araf-tv2* overexpression blocked Ras activation of endogenous Erk1/2 in embryos. Embryos at the one-cell stage were injected with zebrafish *kras G12V* mRNA (*F*) or *nras G12V* (*G*) mRNA alone or in combination with different doses of *araf-tv2* mRNA and harvested at 75% epiboly stage for immunoblotting. The relative levels of p-Erk1/2 were quantified. *H* and *I, araf-tv2* overexpression inhibited ectopic Erk1/2 activation by *kras G12V* (*H*) or *nras G12V* (*I*) overexpression in embryos. The injected embryos at the shield stage were subjected to immunofluorescence using anti-p-Erk1/2 antibody. The ratio of affected embryos was indicated. *Scale bar*, 200 μm.

Fgf ligands can transduce the signal to Ras/MAPK (20). Given that Araf-tv2 retains the Ras-binding domain, we assumed that it could associate with Ras proteins. Immunoprecipitation results in HEK293T cells showed that zebrafish Araf-tv2 associated with human KRAS and NRAS and much more strongly with their constitutively active forms (KRAS G12V and NRAS G12V) (Fig. 6*A*). However, Araf-tv2 appeared not interacting with human HRAS or HRAS G12V, which correlated with the previous report that Araf protein has low affinity with Hras (21).

We then tested whether association of Araf-tv2 with Ras would disrupt Ras-Raf interaction. We found that KRAS G12V could interact strongly with Araf-tv1 and Raf1a and weakly with Braf in HEK293T cells. The physical association between KRAS G12V and full-length Raf proteins was markedly reduced by co-transfection of Araf-tv2 (Fig. 6*B*), suggesting an antagonizing effect on Ras-Raf binding. Further biochemical analyses revealed that Araf-tv2 overexpression in HEK293T cells almost abolished KRAS G12V- or NRAS-induced p-ERK1/2 and reduced Hras-induced p-ERK1/2 to a lesser extent (Fig. 6, *C–E*). In contrast, Araf-tv1 overexpression enhanced ERK1/2 activation by various RAS proteins to some degrees. These results indicate that Araf-tv2 attenuates MAPK activation by blocking transduction of Ras signals to Raf kinases.

The antagonizing effect of *araf-tv2* on Ras/MAPK signaling was further substantiated in zebrafish embryos. Injection of *in vitro* synthesized zebrafish *kras G12V* or *nras G12V* mRNA could increase the amount of p-Erk1/2 (Fig. 6, *F* and *G*) and induce ectopic activation of p-Erk1/2 in zebrafish embryos (Fig. 6, *H* and *I*), which were effectively inhibited by co-injection of *araf-tv2* mRNA. Thus, Araf-tv2 is truly a direct antagonist of Ras and acts to block its downstream MAPK activation.

##### Ras-induced Developmental Defects Are Counteracted by Araf-tv2

**FIGURE 7.**
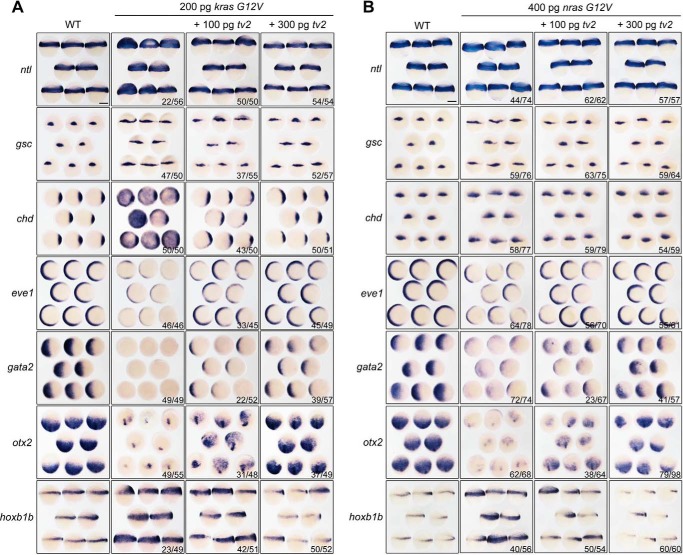
**araf-tv2 overexpression antagonizes *ras*-induced marker changes.** *A* and *B*, expression patterns of marker genes. Embryos at the one-cell stage were injected with zebrafish *kras G12V* (*A*) or *nras G12V* (*B*) mRNA alone or in combination with increasing doses of *araf-tv2*, and harvested for whole-mount *in situ* at the shield stage for *ntl*, *gsc*, *chd*, *eve1*, and *gata2* or at the 75% epiboly stage for *otx2* and *hoxb1b.* Orientations of embryos: animal-pole views with dorsal to the right for *chd*, *eve1*, and *gata2*; lateral views for *ntl* and *hoxb1b*; dorsal views for *gsc*; animal-pole views with dorsal to the bottom for *otx2*. The ratio of embryos with marker change was indicated. *Scale bar*, 200 μm.

We next investigated developmental effect of *ras* overexpression by injecting zebrafish *kras G12V* or *nras G12V* mRNA into one-cell stage embryos and examining the expression of germ layer markers at gastrulation stages. Results showed that overexpression of *ras* mRNA led to up-regulated expression of *ntl*, *gsc*, *chd*, and *hoxb1b* with a reduction of *eve1*, *gata2*, and *otx2* (Fig. 7, *A* and *B*). The high degree of similarity in change of markers expression patterns between *fgf17b* (Fig. 5*A*) and *ras* overexpression indicates that Ras indeed mediates Fgf signaling for regulating embryonic development. Importantly, those effects of *ras* overexpression on the markers expression were alleviated by co-overexpression of *araf-tv2* mRNA (Fig. 7, *A* and *B*). It is speculated that endogenous Araf-tv2 might act to restrict Fgf/Ras signaling activity during embryonic development.

**FIGURE 8.**
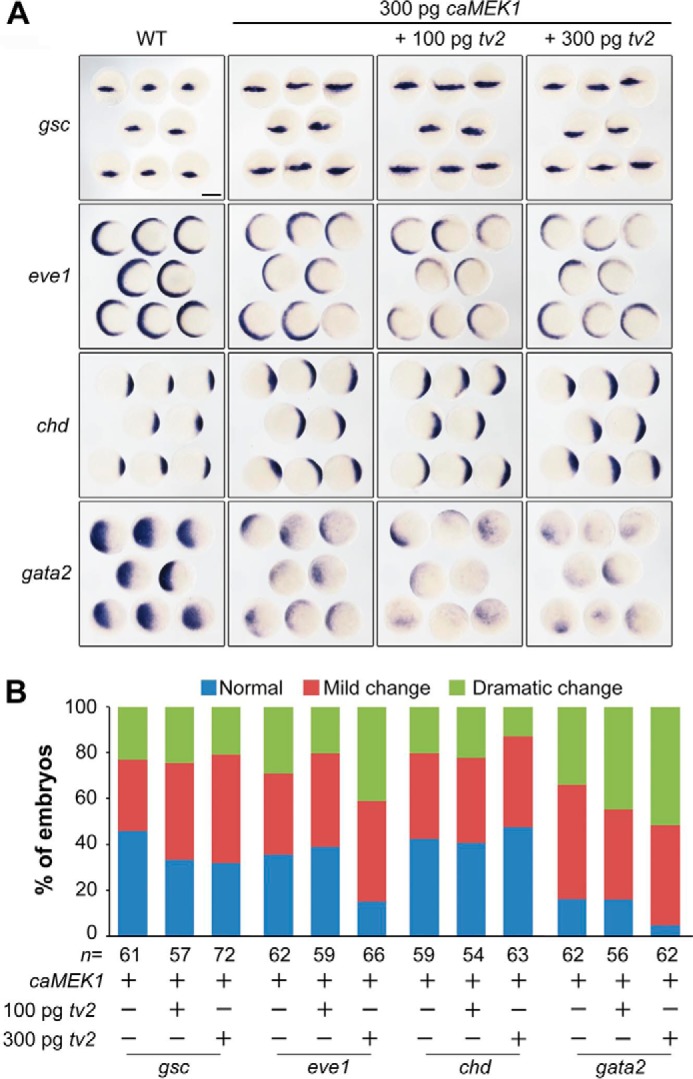
**araf-tv2 overexpression has no effect on caMEK1-induced marker changes.** Embryos at the one-cell stage were injected with *caMEK1* mRNA alone or together with *araf-tv2* mRNA and examined for marker expression at the shield stage. *A*, expression patterns of marker genes. For *chd*, *eve1*, and *gata2*, embryos were shown in animal-pole view with dorsal to the right; for *gsc*, embryos were orientated in dorsal view. *Scale bar*, 200 μm. *B*, statistical data. *n*, the total number of observed embryos.

Mek1/2 are direct substrates of Raf kinases and phosphorylated Mek1/2 activate downstream Erk1/2. Therefore, it would be expected that overexpression effect of Mek1/2 in embryos will not be affected by *araf-tv2*. We found that injection of human *caMEK1* mRNA encoding a constitutively active form of MEK1 expanded *gsc* and *chd* expression and decreased *eve1* and *gata2* expression (Fig. 8, *A* and *B*), a dorsalized phenotype. These changes were not compromised by co-injection of *araf-tv2* mRNA. This result supports the idea that Araf-tv2 antagonizes Ras signaling upstream Mek1/2.

## Discussion

In this study, we demonstrate the expression of the *araf* splicing variant *araf-tv2* in zebrafish embryos that encodes C-terminally truncated Araf isoform without the kinase domain. Although Araf-tv2 physically binds to Araf-tv1, it has little effect on Araf-tv1 function in mediating Ras/MAPK signaling or repressing TGF-β/Smad2 signaling. We uncover that Araf-tv2 associates with and prevents Ras proteins from signaling to MAPK. In the zebrafish embryo, *araf-tv2* overexpression is sufficient to restrict Fgf/MAPK signaling to regulate germ layer formation and patterning.

The full-length Araf (Araf-tv1), like Braf and Raf1, is well known to transduce Ras signaling to Mek/MAPK (22). We previously uncovered that the full-length Araf attenuates Tgf-β/Smad2 signaling in a kinase activity-dependent fashion (15). Previous studies have demonstrated that the kinase activity of Raf proteins is self-inhibited due to association of the C-terminal kinase domain by the N-terminal regulatory domain (22). We show that co-transfection of Araf-tv2 with Araf-tv1 in mammalian cells neither affects the amount of Araf-tv1-activated p-ERK2 (Fig. 2*F*) nor decelerates Araf-tv1-promoted p-Smad2 degradation (Fig. 2*H*). Probably, intermolecular Araf-tv1/Araf-tv2 complexes take a conformation different from intramolecular Araf-tv1 structure, allowing induction and implementation of the kinase activity of Araf-tv1 in the complexes. We establish that Araf-tv2 strongly interacts with Nras and Kras and blocks the downstream MAPK activation (Fig. 6). Thus, the *araf* locus of the zebrafish genome expresses to produce at least two Araf protein isoforms with distinct signaling functions: full-length Araf to relay Ras signals to Mek/MAPK and to control TGF-β/Smad signaling; Araf-tv2 to block Ras/MAPK signaling.

Both *araf-tv1* and *araf-tv2* are expressed during early development of zebrafish embryos. However, the amount of *araf-tv2* transcripts is very low during cleavage period compared with consistent high levels of *araf-tv1* (Fig. 1*C*). Their functional importance should be different depending on developmental stages. At present, lack of isoform-specific antibodies disallows us to look at spatial distribution of endogenous Araf-tv1 and Araf-tv2 proteins.

We found that *araf-tv2* overexpression is sufficient to inhibit Fgf/MAPK signaling during germ layer formation and patterning (Figs. 3 and 5), which could explain why knockdown of *araf* resulted in excess mesendodermal cells and embryonic dorsalization in the zebrafish embryos (15). One would argue that the defects in *araf* morphants are mainly attributed to loss or reduction of Araf-tv1 instead of Araf-tv2 because *araf* morphants show an increase of p-Smad2 and little change in p-Erk1/2 level. However, unaltered p-Erk1/2 levels in *araf* morphants might be a combined effect of *araf-tv1* and *araf-tv2* loss: loss of *araf-tv1* impairs Mek/MAPK activation to a certain degree and loss of *araf-tv2* enhances Ras/MAPK signaling. Nevertheless, *araf-tv1* and *araf-tv2* appear to take part in germ layer formation and patterning by regulating distinct signaling pathways. The specific developmental functions of *araf* transcript variants need to be verified by genetic depletion of individual variants. We tried to knock out the *araf-tv2*-specfic coding sequence (84 bp) by Cas9 technology, but it was unsuccessful. This effort should continue in the future.

## Author Contributions

C. X. designed the study, performed and analyzed experiments, and wrote the paper. X. L. assisted experiments. A. M. conceived the study and wrote the paper. All authors reviewed the results and approved the final version of the manuscript.

## Footnotes

2 The abbreviations used are:

FGF: fibroblast growth factor
TGF: transforming growth factor
MAPK: mitogen-activated protein kinase
Erk: extracellular signal-regulated kinase.
